# Crystal structure of (*N* ^1^,*N* ^3^-bis­{[1-(4-meth­oxy­benz­yl)-1*H*-1,2,3-triazol-4-yl]methyl­idene}-2,2-di­meth­yl­propane-1,3-di­amine)­bis­(thio­cyanato)­iron(II)

**DOI:** 10.1107/S2056989021003662

**Published:** 2021-04-09

**Authors:** Kateryna Znovjyak, Maksym Seredyuk, Sergey O. Malinkin, Iryna A. Golenya, Tatiana Y. Sliva, Sergiu Shova, Nurullo U. Mulloev

**Affiliations:** a Department of Chemistry, Taras Shevchenko National University of Kyiv, Volodymyrska Street 64, Kyiv, 01601, Ukraine; b Department of Inorganic Polymers, "Petru Poni" Institute of Macromolecular, Chemistry, Romanian Academy of Science, Aleea Grigore Ghica Voda 41-A, Iasi, 700487, Romania; c The Faculty of Physics, Tajik National University, Rudaki Avenue 17, Dushanbe, 734025, Tajikistan

**Keywords:** iron(II) complex, thio­cyanate complex, high-spin state, trigonal distortion, magnetism, crystal structure

## Abstract

The unit cell of the title compound, [Fe^II^(NCS)_2_(C_29_H_32_N_8_O_2_)], consists of eight charge-neutral complex mol­ecules. In the complex mol­ecule, the tetra­dentate ligand *N* ^1^,*N* ^3^-bis­{[1-(4-meth­oxy­benz­yl)-1*H*-1,2,3-triazol-4-yl]methyl­ene}-2,2-di­methyl­propane-1,3-di­amine coordinates to the Fe^II^ ion through the N atoms of the 1,2,3-triazole and aldimine groups. Two thio­cyanate anions, coordinated through their N atoms, complete the coordination sphere of the central Fe ion. In the crystal, neighbouring mol­ecules are linked through weak C⋯C, C⋯N and C⋯S inter­actions into a one-dimensional chain running parallel to [010]. The inter­molecular contacts were qu­anti­fied using Hirshfeld surface analysis and two-dimensional fingerprint plots, revealing the relative contributions of the contacts to the crystal packing to be H⋯H (37.5%), H⋯C/C⋯H (24.7%), H⋯S/S⋯H (15.7%) and H⋯N/N⋯H (11.7%). The average Fe—N bond distance is 2.167 Å, indicating the high-spin state of the Fe^II^ ion, which does not change upon cooling, as demonstrated by low-temperature magnetic susceptibility measurements.

## Abstract 2

The title charge-neutral complex shows a *cis*-arrangement of the thio­cyanate anions, with a severely distorted coordination polyhedron. The one-dimensional supra­molecular architecture of the lattice is formed by weak C⋯C/S/N inter­actions and weak C—H⋯O/C/S/N hydrogen bonds.

## Chemical context   

Fe^II^ complexes based on Schiff bases derived from *N*-substituted 1,2,3-triazole aldehydes represent an inter­esting class of coordination compounds exhibiting spin-state switching between low- and high-spin states in different temperature regions (Hagiwara *et al.*, 2014 ▸, 2016 ▸, 2020 ▸; Hora & Hagiwara, 2017 ▸). In all of the charge-neutral mononuclear complexes of this kind described so far, the thio­cyanate anions occupy the axial position in the coordination sphere and thus are in a *trans*-configuration (Hagiwara & Okada, 2016 ▸; Hagiwara *et al.*, 2017 ▸).
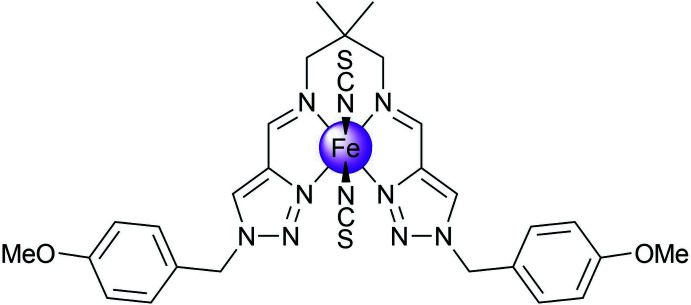


Having ongoing inter­est in functional 3*d*-metal complexes formed by polydentate ligands (Seredyuk *et al.*, 2006 ▸, 2007 ▸, 2011 ▸, 2015 ▸, 2016 ▸; Seredyuk, 2012 ▸; Valverde-Muñoz *et al.*, 2020 ▸), we report here the synthesis and crystal structure of a new Fe^II^ complex based on the tetra­dentate ligand *N* ^1^,*N* ^3^-bis­{[1-(4-meth­oxy­benz­yl)-1*H*-1,2,3-triazol-4-yl]methyl­ene}-2,2-di­methyl­propane-1,3-di­amine with thio­cyanate anions arranged around the iron(II) atom in a *cis*-configuration.

## Structural commentary   

**Figure 1:**
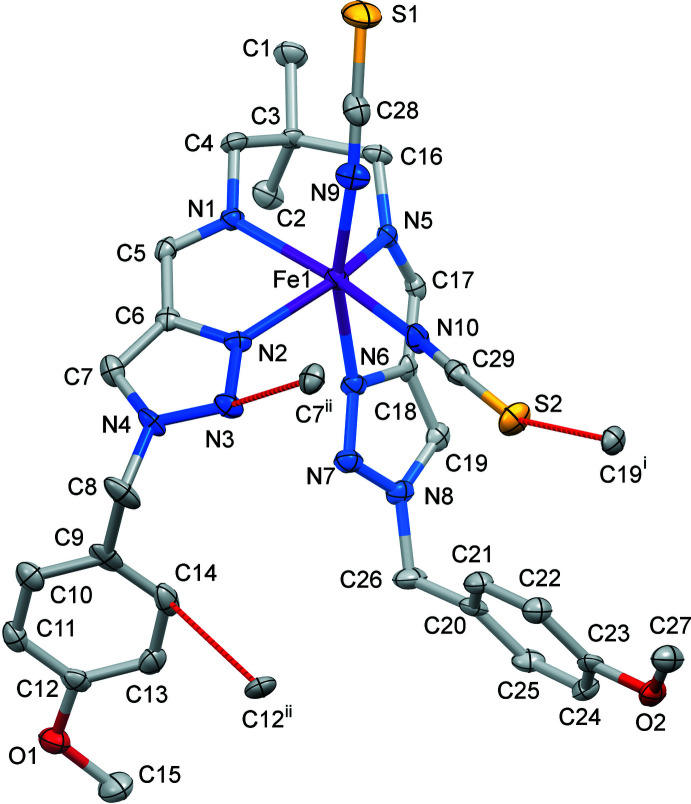
The mol­ecular structure of the title compound with displacement ellipsoids drawn at the 50% probability level. H atoms have been omitted for clarity. Weak inter­molecular element⋯element contacts are represented by dashed red lines.

The Fe^II^ ion of the title complex has a distorted trigonal–prismatic N_6_ coordination environment formed by the four N atoms of the tetra­dentate Schiff-base ligand and the two NCS^−^ counter-ions (Fig. 1 ▸). The average bond length, <Fe—N> = 2.167 Å, is typical for high-spin complexes with an [FeN_6_] chromophore (Gütlich & Goodwin, 2004 ▸). The N—Fe—N angle between the *cis*-aligned thio­cyanate N atoms is 91.6 (1)°. The average trigonal distortion parameters, *Σ* = Σ_1_^12^(|90 − *φ*_i_|), where *φ*_i_ is the angle N—Fe—N′ (Drew *et al.*, 1995 ▸) and *Θ* = Σ_1_^24^(|60 − *θ*_i_|), where *θ*_i_ is the angle generated by the superposition of two opposite faces of an octa­hedron (Chang *et al.*, 1990 ▸), are 127.4 and 481.9°, respectively. The values reveal a great deviation of the coordination environment from an ideal octa­hedron (where *Σ* = *Θ* = 0), and are significantly larger than those of similar [FeN_6_] high-spin *trans*-complexes (Hagiwara *et al.*, 2017 ▸). With the aid of continuous shape measurements (CShM), the shape closest to the Fe-based coordination polyhedron and its distortion can be determined numerically (Kershaw Cook *et al.*, 2015 ▸). The calculated CShM value relative to ideal *O_h_* symmetry is 4.269, and 5.671 relative to ideal *D*_3*h*_ trigonal–prismatic symmetry. Hence, the coordination polyhedron is closer to the former geometry, but is appreciably distorted, as indicated by the calculated value (for an ideal polyhedron CShM = 0). The volume of the [FeN_6_] coordination polyhedron is 12.50 Å^3^.

## Supra­molecular features   

**Figure 2:**
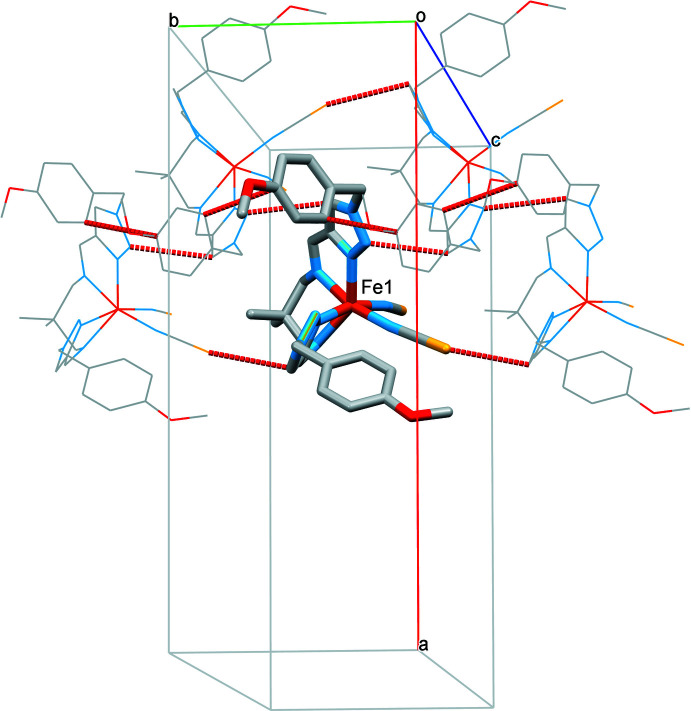
The packing of mol­ecules into one-dimensional chains running parallel to [010] held together by weak C⋯C/N/S bonding.

In the crystal, neighbouring complex mol­ecules form one-dimensional supra­molecular chains propagating parallel to [010] through weak contacts [S2⋯C19^i^ = 3.271 (3) Å, N3⋯C7^ii^ = 3.161 (3) Å and C14⋯C12^ii^ = 3.320 (3) Å; symmetry codes: (i) *x*, −1 + *y*, *z*; (ii) 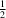 − *x*, −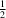 + *y*, *z*] (Fig. 2 ▸). Weak C—H⋯*X* hydrogen bonds (Table 1 ▸) link the chains into a three-dimensional network. No strong hydrogen-bonding or stacking inter­actions are observed between the complex mol­ecules in the crystal structure.

## Hirshfeld surface and 2D fingerprint plots   

**Figure 3:**
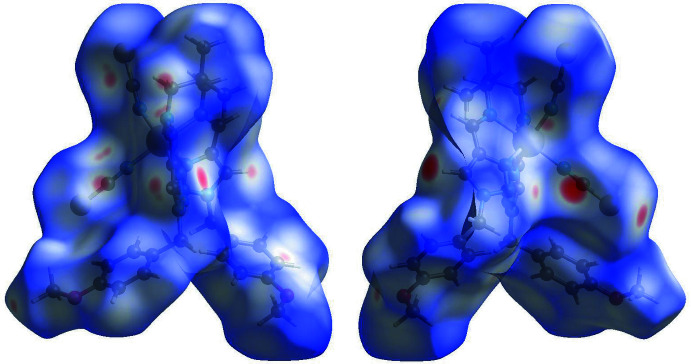
Two projections of *d*_norm_ mapped on Hirshfeld surfaces, showing the inter­actions between molecules. Red areas represent regions where contacts are shorter than the sum of the van der Waals radii, blue areas represent regions where contacts are larger than the sum of van der Waals radii, and white areas are regions where contacts are close to the sum of van der Waals radii.

**Figure 4:**
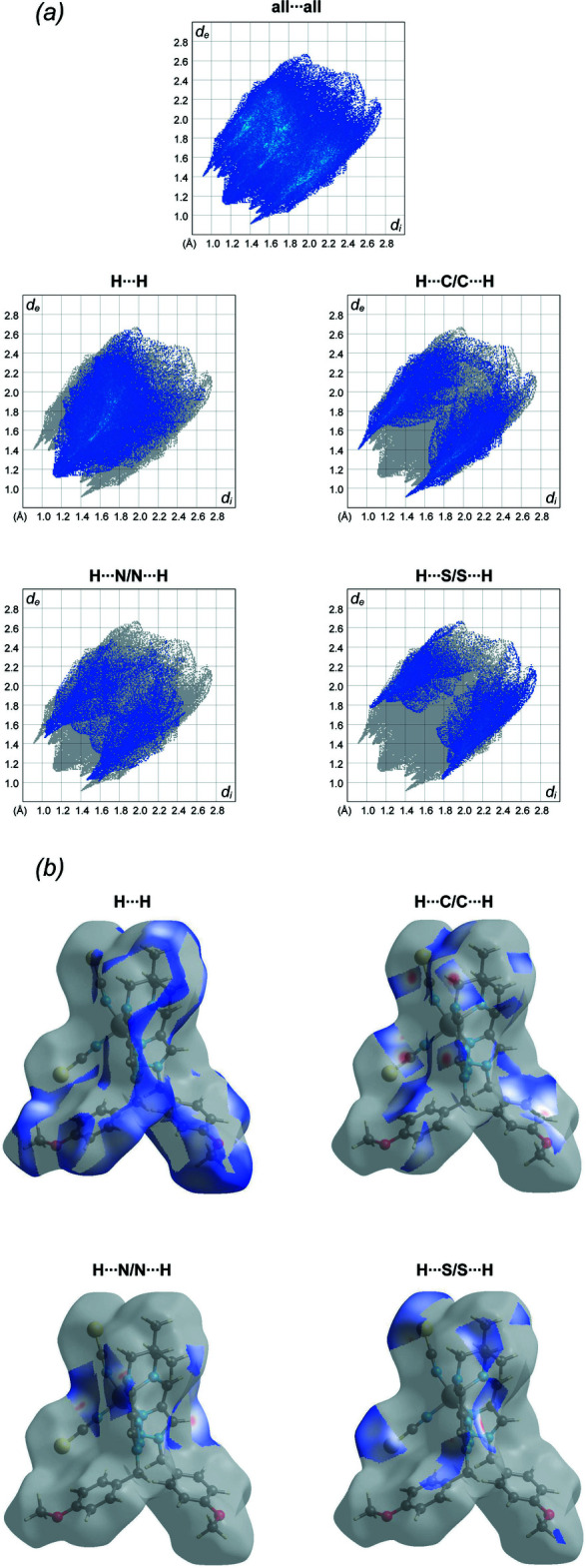
(*a*) The overall two-dimensional fingerprint plot and those delineated into specified inter­actions. (*b*) Hirshfeld surface representations with the function *d*_norm_ plotted onto the surface for the different inter­actions.

Hirshfeld surface analysis was performed and the associated two-dimensional fingerprint plots were generated using *Crystal Explorer* (Turner *et al.*, 2017 ▸), with a standard resolution of the three-dimensional *d*_norm_ surfaces plotted over a fixed colour scale of −0.3171 (red) to 1.6637 (blue) a.u. (Fig. 3 ▸). The pale-red spots symbolize short contacts and negative *d*_norm_ values on the surface correspond to the inter­actions described above. The Hirshfeld surfaces mapped over *d*_norm_ are shown for the H⋯H, H⋯C/C⋯H, H⋯S/S⋯H, and H⋯N/N⋯H contacts, and the two-dimensional fingerprint plots are presented in Fig. 4 ▸, associated with their relative contributions to the Hirshfeld surface. At 37.5%, the largest contribution to the overall crystal packing is from H⋯H inter­actions, which are located in the middle region of the fingerprint plot. H⋯C/C⋯H contacts contribute 24.7%, and the H⋯S/S⋯H contacts contribute 15.7% to the Hirshfeld surface, both resulting in a pair of characteristic wings. The H⋯N/N⋯H contacts, represented by a pair of sharp spikes in the fingerprint plot, make a 11.7% contribution to the Hirshfeld surface.

## Magnetic properties   

**Figure 5:**
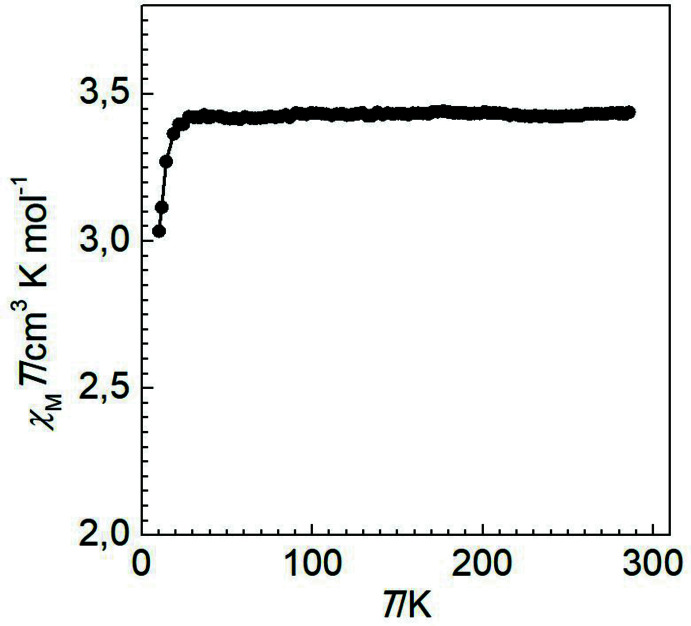
*χ*_M_ *T versus T* plot for the title compound.

Variable-temperature magnetic susceptibility measurements were performed on single crystals (10 mg) of the title compound using a Quantum Design MPMS2 superconducting quantum inter­ference device (SQUID) susceptometer operating at 1 T in the temperature range 10–400 K. Experimental susceptibilities were corrected for the diamagnetism of the holder (gelatine capsule) and of the constituent atoms by the application of Pascal’s constants. The magnetic behaviour of the compound is shown in Fig. 5 ▸ in the form of *χ*_M_ *T* versus *T* (*χ*_M_ is the molar magnetic susceptibility and *T* is the temperature). At 300 K, the *χ*_M_ *T* value is close to 3.40 cm^3^ K mol^−1^, and on cooling the value remains constant down to 30 K. The decrease in *χ*_M_ *T* below 30 K is attributed to the zero-field splitting of the high-spin (*S* = 2) Fe^II^ centres (Kahn, 1993 ▸), which corroborates well with the observed long average Fe—N bond length and the large geometric distortion of the coordination polyhedron of the central Fe^II^ ion.

## Database survey   

A search of the Cambridge Structural Database (CSD, online) reveals five similar Fe^II^ thio­cyanate complexes: derivatives of 1,3-di­amine and *N*-substituted 1,2,3-triazole aldehydes: DURXEV, ADAQUU, ADAREF and solvatomorphs ADAROP and ADARUV (Hagiwara *et al.*, 2017 ▸; Hagiwara & Okada, 2016 ▸). These complexes show hysteretic spin crossover with variation of the Fe—N distances in the range 1.931–1.959 Å for the low-spin state and 2.154–2.169 Å for the high-spin state of the Fe^II^ ions. The reported pseudo-trigonal–prismatic complexes with an [FeN_6_] chromophore are formed by structurally hindered rigid hexa­dentate ligands favouring a trigonal geometry of the central Fe^II^ ion: CABLOH (Voloshin *et al.*, 2001 ▸), BUNSAF (El Hajj *et al.*, 2009 ▸), OWIHAE (Seredyuk *et al.*, 2011 ▸), OTANOO (Stock *et al.*, 2016 ▸). The complex CUWQAP, recently reported by us (Znovjyak *et al.*, 2020 ▸), has a similar strongly distorted coordination environment of the central Fe^II^ ion. Table 2 ▸ collates the distortion parameters *Σ*, *Θ* and CShM for the pseudo-trigonal–prismatic complexes mentioned above.

## Synthesis and crystallization   

The ligand of the title compound was obtained *in situ* by condensation of 2,2-dimethyl-1,3-propanedi­amine (24 µL, 0.20 mmol) with 1-(4-meth­oxy­benz­yl)-1*H*-1,2,3-triazole-4-carbaldehyde (92 mg, 0.45 mmol) by boiling in methanol for 5 min and was subsequently reacted with [Fe(py)_4_(NCS)_2_] (100 mg, 0.20 mmol) and ascorbic acid (11 mg, 0.06 mmol) dissolved in a minimum of boiling methanol. The yellow solution formed was slowly cooled to ambient temperature. Yellow–orange crystals then precipitated and were filtered off. Elemental analysis calculated (%) for C_29_H_32_FeN_10_O_2_S_2_: C, 51.79; H, 4.80; N, 20.82; S, 9.53. Found: C, 52.02; H, 4.68; N, 20.77; S, 9.40. IR *v* (cm^−1^, KBr): 1614 (C=N), 2070, 2118 (NCS).

## Refinement   

Crystal data, data collection and structure refinement details are summarized in Table 3 ▸. H atoms were positioned geom­etrically (C—H = 0.93–0.97 Å) and refined as riding with *U*_iso_(H) = 1.2*U*_eq_(C) or 1.5*U*_eq_(C-meth­yl).

## Supplementary Material

Crystal structure: contains datablock(s) I. DOI: 10.1107/S2056989021003662/cq2043sup1.cif

Structure factors: contains datablock(s) I. DOI: 10.1107/S2056989021003662/cq2043Isup2.hkl

Supporting information file. DOI: 10.1107/S2056989021003662/cq2043Isup3.cdx

CCDC reference: 2075540

Additional supporting information:  crystallographic information; 3D view; checkCIF report

## Figures and Tables

**Table 1:** Hydrogen-bond geometry (Å, °)

*D*—H⋯*A*	*D*—H	H⋯*A*	*D*⋯*A*	*D*—H⋯*A*
C27—H27*A*⋯O1^i^	0.96	2.60	3.517 (4)	161
C20—H20*B*⋯O2^ii^	0.97	2.60	3.282 (4)	127
C19—H19⋯C28^iii^	0.93	2.75	3.574 (5)	148
C19—H19⋯S1^iii^	0.93	2.98	3.825 (4)	152
C17—H17⋯N10^iii^	0.93	2.67	3.416 (4)	138
C17—H17⋯C29^iii^	0.93	2.85	3.685 (5)	150
C16—H16*A*⋯C29^iii^	0.97	2.73	3.667 (5)	163
C5—H5⋯N9^iv^	0.93	2.67	3.590 (5)	173
C7—H7⋯N10^iv^	0.93	2.75	3.614 (5)	156
C7—H7⋯C29^iv^	0.93	2.49	3.400 (5)	166
C7—H7⋯S2^iv^	0.93	2.99	3.752 (5)	140

Symmetry codes: (i) x+{\script{1\over 2}}, y-1, -z+{\script{1\over 2}}; (ii) -x+1, y+{\script{1\over 2}}, -z+{\script{1\over 2}}; (iii) -x+1, -y+1, -z+1; (iv) -x+{\script{1\over 2}}, y+{\script{1\over 2}}, z.

**Table 2:** Comparison of the distortion parameters (Å, °) for indicated Fe^II^ complexes

	<Fe—N>	Σ	Θ	CShM (*D*_3*h*_)
Title compound	2.167	127.4	481.9	5.671
CUWQAP	2.186	149.38	453.2	4.008
CABLOH	1.899	725.74	178.16	0.525
BUNSAF	2.218	703.65	201.07	1.887
OWIHAE	2.202	894.48	206.57	0.602
OTANOO^*a*^	2.191	697.3	183.24	1.098

Note: (*a*) Parameters averaged over five independent complex cations.

**Table 3:** Experimental details

Crystal data	
Chemical formula	[Fe(NCS)_2_(C_27_H_32_N_8_O_2_)]
*M*_r_	672.61
Crystal system, space group	Orthorhombic, *P* *b* *c* *a*
Temperature (K)	99
*a*, *b*, *c* (Å)	22.8809 (15), 9.0485 (4), 31.2662 (18)
*V* (Å^3^)	6473.3 (6)
*Z*	8
Radiation type	Mo *K*α
μ (mm^−1^)	0.64
Crystal size (mm)	0.3 × 0.2 × 0.05

Data collection	
Diffractometer	Rigaku Oxford Diffraction Xcalibur, Eos
Absorption correction	Multi-scan (*CrysAlis PRO*; Rigaku OD, 2015 ▸)
*T*_min_, *T*_max_	0.983, 1.000
No. of measured, independent and observed [*I* > 2σ(*I*)] reflections	14323, 5718, 4331
*R*_int_	0.062

Refinement	
*R*[*F* ^2^ > 2σ(*F* ^2^)], *wR*(*F* ^2^), *S*	0.059, 0.111, 1.10
No. of reflections	5718
No. of parameters	401
H-atom treatment	H-atom parameters constrained
Δρ_max_, Δρ_min_ (e Å^−3^)	0.45, −0.35

Computer programs: *CrysAlis PRO* (Rigaku OD, 2015 ▸), *SIR2008* (Burla *et al.*, 2007 ▸), *SHELXL2018/3* (Sheldrick, 2015 ▸) and *OLEX2* (Dolomanov *et al.*, 2009 ▸).

## supplementary crystallographic information

### Crystal data

**Table:**

[Fe(NCS)_2_(C_27_H_32_N_8_O_2_)]	*D*_x_ = 1.380 Mg m^−^^3^
*M**_r_* = 672.61	Mo *K*α radiation, λ = 0.71073 Å
Orthorhombic, *P**b**c**a*	Cell parameters from 3410 reflections
*a* = 22.8809 (15) Å	θ = 2.2–26.9°
*b* = 9.0485 (4) Å	µ = 0.64 mm^−^^1^
*c* = 31.2662 (18) Å	*T* = 99 K
*V* = 6473.3 (6) Å^3^	Plate, clear dark red
*Z* = 8	0.3 × 0.2 × 0.05 mm
*F*(000) = 2800	

### Data collection

**Table:**

Rigaku Oxford Diffraction Xcalibur, Eos diffractometer	4331 reflections with *I* > 2σ(*I*)
Detector resolution: 8.0797 pixels mm^-1^	*R*_int_ = 0.062
ω scans	θ_max_ = 25.0°, θ_min_ = 1.8°
Absorption correction: multi-scan (CrysAlisPro; Rigaku OD, 2015)	*h* = −10→27
*T*_min_ = 0.983, *T*_max_ = 1.000	*k* = −10→10
14323 measured reflections	*l* = −35→37
5718 independent reflections	

### Refinement

**Table:**

Refinement on *F*^2^	0 restraints
Least-squares matrix: full	Hydrogen site location: inferred from neighbouring sites
*R*[*F*^2^ > 2σ(*F*^2^)] = 0.059	H-atom parameters constrained
*wR*(*F*^2^) = 0.111	*w* = 1/[σ^2^(*F*_o_^2^) + (0.0332*P*)^2^] where *P* = (*F*_o_^2^ + 2*F*_c_^2^)/3
*S* = 1.10	(Δ/σ)_max_ < 0.001
5718 reflections	Δρ_max_ = 0.45 e Å^−^^3^
401 parameters	Δρ_min_ = −0.35 e Å^−^^3^

### Special details

**Table:**

Geometry. All esds (except the esd in the dihedral angle between two l.s. planes) are estimated using the full covariance matrix. The cell esds are taken into account individually in the estimation of esds in distances, angles and torsion angles; correlations between esds in cell parameters are only used when they are defined by crystal symmetry. An approximate (isotropic) treatment of cell esds is used for estimating esds involving l.s. planes.

### Fractional atomic coordinates and isotropic or equivalent isotropic displacement parameters (Å^2^)

**Table:**

	*x*	*y*	*z*	*U*_iso_*/*U*_eq_	
Fe1	0.36927 (2)	0.44257 (5)	0.48750 (2)	0.01653 (14)	
S1	0.35511 (5)	0.27805 (13)	0.63189 (3)	0.0447 (3)	
S2	0.45693 (4)	0.01869 (10)	0.41509 (3)	0.0291 (3)	
O1	0.21074 (11)	0.7698 (3)	0.22792 (7)	0.0291 (6)	
O2	0.59076 (10)	0.1143 (2)	0.27020 (7)	0.0248 (6)	
N1	0.30621 (12)	0.6015 (3)	0.51744 (8)	0.0178 (7)	
N2	0.29144 (12)	0.4260 (3)	0.44994 (8)	0.0170 (7)	
N3	0.27703 (12)	0.3551 (3)	0.41465 (9)	0.0214 (7)	
N4	0.22437 (12)	0.4080 (3)	0.40340 (9)	0.0206 (7)	
N5	0.42995 (12)	0.6039 (3)	0.51301 (9)	0.0164 (6)	
N6	0.40524 (12)	0.5724 (3)	0.43049 (9)	0.0187 (7)	
N7	0.40240 (13)	0.5591 (3)	0.38880 (9)	0.0219 (7)	
N8	0.45248 (13)	0.6174 (3)	0.37344 (9)	0.0211 (7)	
N9	0.36115 (13)	0.3225 (3)	0.54366 (10)	0.0257 (7)	
N10	0.41108 (12)	0.2661 (3)	0.45691 (9)	0.0227 (7)	
C1	0.37528 (17)	0.8159 (4)	0.60960 (11)	0.0310 (10)	
H1A	0.375354	0.733805	0.629056	0.047*	
H1B	0.342017	0.877495	0.615288	0.047*	
H1C	0.410447	0.872295	0.613305	0.047*	
C3	0.37212 (15)	0.7588 (4)	0.56379 (11)	0.0193 (8)	
C4	0.31474 (15)	0.6730 (4)	0.55938 (10)	0.0227 (9)	
H4A	0.313404	0.597677	0.581427	0.027*	
H4B	0.282487	0.740361	0.564420	0.027*	
C16	0.42617 (15)	0.6583 (4)	0.55718 (10)	0.0196 (8)	
H16A	0.461308	0.713397	0.564006	0.024*	
H16B	0.423858	0.574790	0.576577	0.024*	
C5	0.25706 (15)	0.6127 (4)	0.49871 (10)	0.0185 (8)	
H5	0.227835	0.674579	0.509020	0.022*	
C6	0.24881 (15)	0.5244 (3)	0.46060 (11)	0.0166 (8)	
C7	0.20567 (15)	0.5129 (4)	0.43064 (11)	0.0233 (9)	
H7	0.170935	0.566109	0.429329	0.028*	
C8	0.19663 (18)	0.3560 (4)	0.36374 (11)	0.0319 (10)	
H8A	0.155587	0.336456	0.369061	0.038*	
H8B	0.214826	0.264241	0.354803	0.038*	
C9	0.20226 (17)	0.4690 (4)	0.32836 (11)	0.0252 (9)	
C10	0.15496 (16)	0.5551 (4)	0.31594 (11)	0.0255 (9)	
H10	0.119558	0.544958	0.330250	0.031*	
C11	0.15932 (16)	0.6545 (4)	0.28308 (11)	0.0243 (9)	
H11	0.127045	0.711095	0.275409	0.029*	
C12	0.21154 (16)	0.6713 (4)	0.26122 (11)	0.0227 (9)	
C13	0.26012 (16)	0.5918 (4)	0.27391 (11)	0.0279 (9)	
H13	0.295853	0.605572	0.260294	0.033*	
C14	0.25489 (16)	0.4906 (4)	0.30743 (12)	0.0285 (9)	
H14	0.287484	0.436628	0.315875	0.034*	
C15	0.26393 (17)	0.7909 (5)	0.20445 (12)	0.0402 (11)	
H15A	0.257419	0.860635	0.181795	0.060*	
H15B	0.276332	0.698306	0.192538	0.060*	
H15C	0.293636	0.827953	0.223293	0.060*	
C17	0.46360 (15)	0.6705 (4)	0.48662 (11)	0.0185 (8)	
H17	0.491715	0.737278	0.496004	0.022*	
C18	0.45624 (14)	0.6381 (3)	0.44164 (11)	0.0159 (8)	
C19	0.48706 (16)	0.6670 (3)	0.40516 (11)	0.0201 (8)	
H19	0.523657	0.710946	0.402782	0.024*	
C20	0.46345 (17)	0.6144 (4)	0.32700 (11)	0.0291 (10)	
H20A	0.485754	0.701335	0.319094	0.035*	
H20B	0.426354	0.618584	0.312035	0.035*	
C21	0.49624 (15)	0.4783 (4)	0.31281 (10)	0.0196 (8)	
C22	0.54372 (15)	0.4919 (4)	0.28529 (10)	0.0231 (9)	
H22	0.555641	0.585120	0.276344	0.028*	
C23	0.57315 (15)	0.3689 (4)	0.27121 (11)	0.0230 (9)	
H23	0.604410	0.379555	0.252458	0.028*	
C24	0.55672 (15)	0.2287 (4)	0.28470 (10)	0.0190 (8)	
C25	0.50976 (15)	0.2136 (4)	0.31199 (11)	0.0219 (8)	
H25	0.498300	0.120252	0.321203	0.026*	
C26	0.47962 (16)	0.3382 (4)	0.32569 (11)	0.0232 (9)	
H26	0.447708	0.327227	0.343831	0.028*	
C27	0.58123 (16)	−0.0289 (4)	0.28871 (11)	0.0283 (9)	
H27A	0.611071	−0.095612	0.279086	0.042*	
H27B	0.543625	−0.065338	0.280106	0.042*	
H27C	0.582617	−0.021393	0.319323	0.042*	
C28	0.35849 (16)	0.3024 (4)	0.58016 (13)	0.0249 (9)	
C29	0.42995 (15)	0.1637 (4)	0.43909 (11)	0.0199 (8)	
C2	0.37357 (17)	0.8902 (4)	0.53291 (11)	0.0284 (9)	
H2A	0.410648	0.939228	0.535081	0.043*	
H2B	0.342892	0.958133	0.540118	0.043*	
H2C	0.368063	0.855479	0.504172	0.043*	

### Atomic displacement parameters (Å^2^)

**Table:**

	*U*^11^	*U*^22^	*U*^33^	*U*^12^	*U*^13^	*U*^23^
Fe1	0.0201 (3)	0.0141 (2)	0.0154 (3)	0.0026 (2)	−0.0036 (2)	−0.0026 (2)
S1	0.0471 (7)	0.0629 (8)	0.0239 (6)	−0.0011 (6)	0.0011 (5)	0.0165 (6)
S2	0.0348 (6)	0.0177 (5)	0.0348 (6)	0.0031 (4)	0.0071 (5)	−0.0057 (5)
O1	0.0315 (16)	0.0343 (15)	0.0215 (14)	−0.0001 (13)	−0.0023 (12)	0.0044 (13)
O2	0.0298 (15)	0.0227 (13)	0.0220 (14)	0.0095 (12)	0.0042 (12)	−0.0018 (12)
N1	0.0216 (17)	0.0167 (14)	0.0151 (16)	0.0030 (13)	−0.0001 (13)	−0.0014 (13)
N2	0.0231 (17)	0.0132 (14)	0.0146 (15)	−0.0012 (13)	0.0007 (13)	0.0011 (13)
N3	0.0267 (18)	0.0179 (15)	0.0194 (17)	−0.0017 (14)	−0.0055 (14)	−0.0015 (14)
N4	0.0231 (17)	0.0176 (15)	0.0210 (17)	−0.0022 (13)	−0.0118 (14)	0.0016 (14)
N5	0.0182 (16)	0.0145 (14)	0.0165 (15)	0.0056 (12)	−0.0027 (13)	−0.0039 (14)
N6	0.0243 (17)	0.0171 (15)	0.0148 (16)	0.0042 (13)	−0.0010 (13)	−0.0042 (14)
N7	0.0283 (18)	0.0210 (16)	0.0164 (16)	0.0027 (14)	−0.0008 (14)	−0.0031 (15)
N8	0.0300 (19)	0.0151 (15)	0.0182 (17)	0.0069 (14)	0.0029 (15)	−0.0025 (14)
N9	0.034 (2)	0.0213 (17)	0.0221 (18)	−0.0045 (15)	−0.0018 (16)	0.0002 (15)
N10	0.0229 (17)	0.0190 (16)	0.0263 (18)	0.0049 (14)	−0.0070 (15)	−0.0054 (15)
C1	0.038 (2)	0.033 (2)	0.022 (2)	0.0049 (19)	−0.0067 (19)	−0.0095 (19)
C3	0.025 (2)	0.0200 (18)	0.0134 (18)	0.0065 (17)	−0.0021 (16)	−0.0069 (16)
C4	0.027 (2)	0.028 (2)	0.0133 (19)	0.0065 (17)	0.0028 (16)	−0.0028 (17)
C16	0.027 (2)	0.0182 (18)	0.0134 (19)	−0.0020 (16)	−0.0035 (16)	−0.0020 (16)
C5	0.019 (2)	0.0163 (17)	0.021 (2)	0.0045 (15)	0.0046 (16)	0.0024 (16)
C6	0.0174 (19)	0.0148 (17)	0.0178 (19)	−0.0014 (15)	0.0017 (16)	0.0041 (16)
C7	0.020 (2)	0.0203 (19)	0.029 (2)	0.0009 (16)	−0.0046 (18)	0.0029 (18)
C8	0.045 (3)	0.023 (2)	0.028 (2)	−0.0019 (19)	−0.022 (2)	−0.0017 (19)
C9	0.036 (2)	0.0183 (19)	0.022 (2)	−0.0021 (18)	−0.0166 (18)	−0.0089 (17)
C10	0.024 (2)	0.026 (2)	0.027 (2)	−0.0047 (18)	−0.0077 (17)	−0.0019 (19)
C11	0.022 (2)	0.023 (2)	0.028 (2)	0.0021 (17)	−0.0092 (18)	−0.0017 (18)
C12	0.029 (2)	0.0229 (19)	0.0159 (19)	0.0002 (17)	−0.0064 (18)	−0.0053 (17)
C13	0.027 (2)	0.033 (2)	0.024 (2)	0.0076 (18)	0.0018 (18)	−0.0093 (19)
C14	0.030 (2)	0.024 (2)	0.031 (2)	0.0126 (18)	−0.012 (2)	−0.0089 (19)
C15	0.034 (3)	0.057 (3)	0.029 (2)	−0.003 (2)	0.007 (2)	0.005 (2)
C17	0.0162 (19)	0.0157 (17)	0.024 (2)	0.0031 (15)	−0.0044 (17)	0.0006 (17)
C18	0.0151 (19)	0.0110 (16)	0.022 (2)	0.0042 (15)	−0.0013 (16)	−0.0035 (16)
C19	0.023 (2)	0.0131 (17)	0.025 (2)	0.0050 (16)	0.0023 (17)	0.0012 (17)
C20	0.043 (3)	0.030 (2)	0.014 (2)	0.0088 (19)	0.0047 (18)	0.0024 (18)
C21	0.029 (2)	0.0201 (19)	0.0099 (18)	0.0015 (17)	−0.0038 (16)	0.0009 (16)
C22	0.033 (2)	0.0198 (19)	0.0166 (19)	−0.0028 (17)	0.0020 (18)	0.0039 (17)
C23	0.023 (2)	0.028 (2)	0.018 (2)	0.0018 (17)	0.0064 (17)	−0.0004 (18)
C24	0.024 (2)	0.0229 (19)	0.0105 (18)	0.0024 (17)	−0.0032 (16)	−0.0051 (16)
C25	0.029 (2)	0.0167 (18)	0.020 (2)	0.0011 (17)	0.0007 (17)	−0.0003 (17)
C26	0.026 (2)	0.028 (2)	0.015 (2)	−0.0004 (18)	0.0024 (16)	0.0021 (18)
C27	0.042 (2)	0.021 (2)	0.022 (2)	0.0107 (18)	0.0012 (19)	0.0001 (17)
C28	0.023 (2)	0.0181 (19)	0.034 (2)	−0.0012 (16)	−0.0035 (19)	0.0049 (19)
C29	0.019 (2)	0.0207 (19)	0.020 (2)	−0.0035 (16)	−0.0035 (16)	0.0056 (18)
C2	0.040 (2)	0.0204 (19)	0.025 (2)	0.0086 (18)	−0.0059 (19)	−0.0034 (18)

### Geometric parameters (Å, º)

**Table:**

Fe1—N1	2.242 (3)	C6—C7	1.365 (5)
Fe1—N2	2.138 (3)	C7—H7	0.9300
Fe1—N5	2.167 (3)	C8—H8A	0.9700
Fe1—N6	2.288 (3)	C8—H8B	0.9700
Fe1—N9	2.073 (3)	C8—C9	1.512 (5)
Fe1—N10	2.092 (3)	C9—C10	1.389 (5)
S1—C28	1.634 (4)	C9—C14	1.384 (5)
S2—C29	1.633 (4)	C10—H10	0.9300
O1—C12	1.371 (4)	C10—C11	1.369 (5)
O1—C15	1.434 (4)	C11—H11	0.9300
O2—C24	1.373 (4)	C11—C12	1.385 (5)
O2—C27	1.435 (4)	C12—C13	1.382 (5)
N1—C4	1.475 (4)	C13—H13	0.9300
N1—C5	1.272 (4)	C13—C14	1.397 (5)
N2—N3	1.319 (4)	C14—H14	0.9300
N2—C6	1.362 (4)	C15—H15A	0.9600
N3—N4	1.344 (4)	C15—H15B	0.9600
N4—C7	1.345 (4)	C15—H15C	0.9600
N4—C8	1.470 (4)	C17—H17	0.9300
N5—C16	1.469 (4)	C17—C18	1.447 (4)
N5—C17	1.279 (4)	C18—C19	1.366 (4)
N6—N7	1.311 (4)	C19—H19	0.9300
N6—C18	1.355 (4)	C20—H20A	0.9700
N7—N8	1.350 (4)	C20—H20B	0.9700
N8—C19	1.346 (4)	C20—C21	1.508 (5)
N8—C20	1.474 (4)	C21—C22	1.391 (5)
N9—C28	1.157 (4)	C21—C26	1.384 (5)
N10—C29	1.164 (4)	C22—H22	0.9300
C1—H1A	0.9600	C22—C23	1.373 (5)
C1—H1B	0.9600	C23—H23	0.9300
C1—H1C	0.9600	C23—C24	1.388 (5)
C1—C3	1.524 (4)	C24—C25	1.379 (5)
C3—C4	1.531 (5)	C25—H25	0.9300
C3—C16	1.549 (4)	C25—C26	1.389 (5)
C3—C2	1.532 (5)	C26—H26	0.9300
C4—H4A	0.9700	C27—H27A	0.9600
C4—H4B	0.9700	C27—H27B	0.9600
C16—H16A	0.9700	C27—H27C	0.9600
C16—H16B	0.9700	C2—H2A	0.9600
C5—H5	0.9300	C2—H2B	0.9600
C5—C6	1.447 (5)	C2—H2C	0.9600
			
N1—Fe1—N6	103.14 (10)	H8A—C8—H8B	108.0
N2—Fe1—N1	74.82 (10)	C9—C8—H8A	109.4
N2—Fe1—N5	141.53 (10)	C9—C8—H8B	109.4
N2—Fe1—N6	84.71 (10)	C10—C9—C8	121.2 (4)
N5—Fe1—N1	80.01 (10)	C14—C9—C8	121.1 (3)
N5—Fe1—N6	73.16 (10)	C14—C9—C10	117.8 (3)
N9—Fe1—N1	85.69 (11)	C9—C10—H10	119.3
N9—Fe1—N2	110.72 (11)	C11—C10—C9	121.4 (4)
N9—Fe1—N5	95.66 (11)	C11—C10—H10	119.3
N9—Fe1—N6	163.98 (11)	C10—C11—H11	119.8
N9—Fe1—N10	91.62 (11)	C10—C11—C12	120.3 (3)
N10—Fe1—N1	167.02 (11)	C12—C11—H11	119.8
N10—Fe1—N2	94.38 (11)	O1—C12—C11	115.8 (3)
N10—Fe1—N5	112.91 (10)	O1—C12—C13	124.5 (3)
N10—Fe1—N6	82.61 (10)	C13—C12—C11	119.7 (3)
C12—O1—C15	117.6 (3)	C12—C13—H13	120.4
C24—O2—C27	117.5 (3)	C12—C13—C14	119.2 (4)
C4—N1—Fe1	124.6 (2)	C14—C13—H13	120.4
C5—N1—Fe1	115.3 (2)	C9—C14—C13	121.5 (3)
C5—N1—C4	119.4 (3)	C9—C14—H14	119.3
N3—N2—Fe1	134.6 (2)	C13—C14—H14	119.3
N3—N2—C6	110.1 (3)	O1—C15—H15A	109.5
C6—N2—Fe1	114.6 (2)	O1—C15—H15B	109.5
N2—N3—N4	105.6 (3)	O1—C15—H15C	109.5
N3—N4—C7	111.8 (3)	H15A—C15—H15B	109.5
N3—N4—C8	119.6 (3)	H15A—C15—H15C	109.5
C7—N4—C8	128.5 (3)	H15B—C15—H15C	109.5
C16—N5—Fe1	122.3 (2)	N5—C17—H17	121.3
C17—N5—Fe1	117.8 (2)	N5—C17—C18	117.5 (3)
C17—N5—C16	118.9 (3)	C18—C17—H17	121.3
N7—N6—Fe1	135.2 (2)	N6—C18—C17	116.0 (3)
N7—N6—C18	109.8 (3)	N6—C18—C19	108.3 (3)
C18—N6—Fe1	109.6 (2)	C19—C18—C17	135.5 (3)
N6—N7—N8	106.0 (3)	N8—C19—C18	104.4 (3)
N7—N8—C20	119.2 (3)	N8—C19—H19	127.8
C19—N8—N7	111.5 (3)	C18—C19—H19	127.8
C19—N8—C20	129.2 (3)	N8—C20—H20A	109.0
C28—N9—Fe1	157.3 (3)	N8—C20—H20B	109.0
C29—N10—Fe1	174.6 (3)	N8—C20—C21	112.9 (3)
H1A—C1—H1B	109.5	H20A—C20—H20B	107.8
H1A—C1—H1C	109.5	C21—C20—H20A	109.0
H1B—C1—H1C	109.5	C21—C20—H20B	109.0
C3—C1—H1A	109.5	C22—C21—C20	119.9 (3)
C3—C1—H1B	109.5	C26—C21—C20	121.7 (3)
C3—C1—H1C	109.5	C26—C21—C22	118.4 (3)
C1—C3—C4	107.3 (3)	C21—C22—H22	119.7
C1—C3—C16	106.6 (3)	C23—C22—C21	120.7 (3)
C1—C3—C2	109.2 (3)	C23—C22—H22	119.7
C4—C3—C16	112.0 (3)	C22—C23—H23	119.7
C4—C3—C2	110.8 (3)	C22—C23—C24	120.7 (3)
C2—C3—C16	110.8 (3)	C24—C23—H23	119.7
N1—C4—C3	114.6 (3)	O2—C24—C23	115.8 (3)
N1—C4—H4A	108.6	O2—C24—C25	124.9 (3)
N1—C4—H4B	108.6	C25—C24—C23	119.3 (3)
C3—C4—H4A	108.6	C24—C25—H25	120.1
C3—C4—H4B	108.6	C24—C25—C26	119.8 (3)
H4A—C4—H4B	107.6	C26—C25—H25	120.1
N5—C16—C3	111.7 (3)	C21—C26—C25	121.2 (3)
N5—C16—H16A	109.3	C21—C26—H26	119.4
N5—C16—H16B	109.3	C25—C26—H26	119.4
C3—C16—H16A	109.3	O2—C27—H27A	109.5
C3—C16—H16B	109.3	O2—C27—H27B	109.5
H16A—C16—H16B	107.9	O2—C27—H27C	109.5
N1—C5—H5	121.6	H27A—C27—H27B	109.5
N1—C5—C6	116.8 (3)	H27A—C27—H27C	109.5
C6—C5—H5	121.6	H27B—C27—H27C	109.5
N2—C6—C5	118.0 (3)	N9—C28—S1	178.7 (4)
N2—C6—C7	107.5 (3)	N10—C29—S2	178.7 (3)
C7—C6—C5	134.6 (3)	C3—C2—H2A	109.5
N4—C7—C6	105.0 (3)	C3—C2—H2B	109.5
N4—C7—H7	127.5	C3—C2—H2C	109.5
C6—C7—H7	127.5	H2A—C2—H2B	109.5
N4—C8—H8A	109.4	H2A—C2—H2C	109.5
N4—C8—H8B	109.4	H2B—C2—H2C	109.5
N4—C8—C9	111.3 (3)		
			
Fe1—N1—C4—C3	55.4 (4)	C4—C3—C16—N5	66.6 (4)
Fe1—N1—C5—C6	−1.1 (4)	C16—N5—C17—C18	−165.6 (3)
Fe1—N2—N3—N4	−170.5 (2)	C16—C3—C4—N1	−59.6 (4)
Fe1—N2—C6—C5	−8.2 (4)	C5—N1—C4—C3	−134.5 (3)
Fe1—N2—C6—C7	172.3 (2)	C5—C6—C7—N4	−179.2 (4)
Fe1—N5—C16—C3	−70.4 (3)	C6—N2—N3—N4	−0.9 (3)
Fe1—N5—C17—C18	3.1 (4)	C7—N4—C8—C9	73.0 (5)
Fe1—N6—N7—N8	−150.2 (2)	C8—N4—C7—C6	−176.5 (3)
Fe1—N6—C18—C17	−26.4 (3)	C8—C9—C10—C11	−178.1 (3)
Fe1—N6—C18—C19	157.9 (2)	C8—C9—C14—C13	178.2 (3)
O1—C12—C13—C14	−177.7 (3)	C9—C10—C11—C12	0.2 (5)
O2—C24—C25—C26	177.8 (3)	C10—C9—C14—C13	−2.2 (5)
N1—C5—C6—N2	6.2 (5)	C10—C11—C12—O1	177.8 (3)
N1—C5—C6—C7	−174.5 (4)	C10—C11—C12—C13	−2.8 (5)
N2—N3—N4—C7	1.0 (4)	C11—C12—C13—C14	2.9 (5)
N2—N3—N4—C8	177.2 (3)	C12—C13—C14—C9	−0.4 (5)
N2—C6—C7—N4	0.1 (4)	C14—C9—C10—C11	2.3 (5)
N3—N2—C6—C5	179.9 (3)	C15—O1—C12—C11	179.9 (3)
N3—N2—C6—C7	0.5 (4)	C15—O1—C12—C13	0.5 (5)
N3—N4—C7—C6	−0.7 (4)	C17—N5—C16—C3	97.8 (3)
N3—N4—C8—C9	−102.6 (4)	C17—C18—C19—N8	−174.1 (3)
N4—C8—C9—C10	−104.9 (4)	C18—N6—N7—N8	0.0 (3)
N4—C8—C9—C14	74.7 (4)	C19—N8—C20—C21	86.0 (4)
N5—C17—C18—N6	17.1 (4)	C20—N8—C19—C18	−177.8 (3)
N5—C17—C18—C19	−168.7 (4)	C20—C21—C22—C23	−178.4 (3)
N6—N7—N8—C19	0.2 (3)	C20—C21—C26—C25	179.2 (3)
N6—N7—N8—C20	177.9 (3)	C21—C22—C23—C24	−1.0 (5)
N6—C18—C19—N8	0.4 (4)	C22—C21—C26—C25	0.7 (5)
N7—N6—C18—C17	175.4 (3)	C22—C23—C24—O2	−177.1 (3)
N7—N6—C18—C19	−0.3 (4)	C22—C23—C24—C25	1.0 (5)
N7—N8—C19—C18	−0.4 (4)	C23—C24—C25—C26	−0.1 (5)
N7—N8—C20—C21	−91.3 (4)	C24—C25—C26—C21	−0.7 (5)
N8—C20—C21—C22	−133.2 (3)	C26—C21—C22—C23	0.2 (5)
N8—C20—C21—C26	48.3 (5)	C27—O2—C24—C23	170.2 (3)
C1—C3—C4—N1	−176.3 (3)	C27—O2—C24—C25	−7.8 (5)
C1—C3—C16—N5	−176.3 (3)	C2—C3—C4—N1	64.6 (4)
C4—N1—C5—C6	−172.0 (3)	C2—C3—C16—N5	−57.7 (4)

### Hydrogen-bond geometry (Å, º)

**Table:**

*D*—H···*A*	*D*—H	H···*A*	*D*···*A*	*D*—H···*A*
C27—H27*A*···O1^i^	0.96	2.60	3.517 (4)	161
C20—H20*B*···O2^ii^	0.97	2.60	3.282 (4)	127
C19—H19···C28^iii^	0.93	2.75	3.574 (5)	148
C19—H19···S1^iii^	0.93	2.98	3.825 (4)	152
C17—H17···N10^iii^	0.93	2.67	3.416 (4)	138
C17—H17···C29^iii^	0.93	2.85	3.685 (5)	150
C16—H16*A*···C29^iii^	0.97	2.73	3.667 (5)	163
C5—H5···N9^iv^	0.93	2.67	3.590 (5)	173
C7—H7···N10^iv^	0.93	2.75	3.614 (5)	156
C7—H7···C29^iv^	0.93	2.49	3.400 (5)	166
C7—H7···S2^iv^	0.93	2.99	3.752 (5)	140

Symmetry codes: (i) *x*+1/2, *y*−1, −*z*+1/2; (ii) −*x*+1, *y*+1/2, −*z*+1/2; (iii) −*x*+1, −*y*+1, −*z*+1; (iv) −*x*+1/2, *y*+1/2, *z*.
